# Prevalence and sociodemographic predictors of multiple non-communicable diseases risk behaviours among in-school adolescents in Delta State, Nigeria: A cross-sectional study

**DOI:** 10.4314/ahs.v25i2.14

**Published:** 2025-06

**Authors:** Patrick Oyibo, Ejiroghene Martha Umuerri, Nyemike Simeon Awunor, Iyabo Aduke Oyibo, Mamodesan Tudjegbe Okumagba

**Affiliations:** 1 Health Services Research and Management Division, School of Health and Psychological Sciences City, University of London, United Kingdom; 2 Department of Community Medicine, Faculty of Clinical Medicine, College of Health Sciences, Delta State University, Abraka, Nigeria; 3 Department of Medicine, Faculty of Clinical Medicine, College of Health Sciences, Delta State University, Abraka, Nigeria; 4 Department of Paediatrics, Princess of Wales Hospital, Bridgend, United Kingdom

**Keywords:** Non-communicable diseases, Risk behaviours, Clusters, Simultaneous presence, In-school adolescents

## Abstract

**Background:**

NCDs risk behaviours are modifiable and particularly patterned during adolescence. This study assessed the prevalence and sociodemographic predictors of multiple NCDs risk behaviours among in-school adolescents.

**Methods:**

A cross-sectional study design was employed to assess the simultaneous occurrence of NCD risk behaviours among a random multistage sample of 607 participants. Data was collected using an interviewer-administered semi-structured questionnaire. Bivariate and multivariate analysis was carried using the IBM SPSS version 22 software.

**Results:**

The mean age of the study participants was 14.7 (SD = 1.52) years. The prevalence of two and at least three co-occurring NCDs risk behaviours among the study participants 46.1 % (n=280) and 16.6 % (n = 101). Increasing age (AOR=1.84; 95% CI: 1.11 - 3.05), male sex (AOR=1.75; 95% CI: 1.28 - 2.82) and being an urban resident (AOR=1.41; 95% CI: 1.06 - 2.86) were predictors of at least three co-occurring NCDs risk behaviours.

**Conclusion:**

The prevalence of multiple NCDs risk behaviours was relatively high among the study participants. This calls for urgent implementation of interventions at all ecological levels that will equip in-school adolescents with the skills to adopt healthy lifestyles and choices.

## Introduction

Globally, Non-Communicable Diseases (NCDs) are a growing cause of public health concern with catastrophic economic and health burdens^1^,^2^. Over seventy per cent of global deaths annually (41 million) are attributable to NCDs, with over three-quarters of these annual deaths (31.4 million) occurring in low and middle-resource regions of the world^2^. Cardiovascular diseases, chronic airway diseases, diabetes mellitus, and cancers are the commonest NCDs, and they account for over eighty per cent of cases globally^2^.

Although some risk factors for NCDs, like advancing age and genetics, are non-modifiable, many others, such as physical inactivity, tobacco smoking, harmful use of alcohol, and intake of unhealthy diets, are modifiable^3^,^4^. Annually, tobacco smoking and inadequate physical activity account for over 8 million deaths and 830,000 deaths, respectively. Similarly, excess salt intake and alcohol use account for 1.8 million deaths and 3 million deaths yearly, respectively^2^. These modifiable risk factors could occur singly or co-occur in individuals^5^-^7^. The co-occurrence of NCD risk behaviours in individuals exponentially increases their chance of acquiring NCDs^2^. The scientific rationale for preventing and controlling major NCD risk factors is well documented. Evidence shows that reducing the behavioural risk factors will tremendously decrease the prevalence of NCDS^2^-^4^.

The patterns of NCD risk behaviours are potentially shaped during the adolescence period and tend to persist in adulthood^8^. This period is a significant phase of life characterised by the development of mental capacity and significant physical, emotional, and behavioural changes^8^. Over 60% of premature mortalities in adults are linked to NCDs risk behaviours adopted in the adolescence period of life^9^.

More than four-fifths of the global adolescent population are insufficiently physically active^10^. In sub-Saharan Africa (SSA), the prevalence of insufficient physical activity particularly among male adolescents is ranked second highest in the World Bank regions^10^. In Nigeria, between 38.0 % and 53.8 % of in-school adolescents have been reported from previous studies to be insufficiently physical active^11^,^12^. Tobacco is a major preventable cause of illness and death. However, the global prevalence of tobacco smoking among adolescents remain substantial^13^. In Nigeria, a systematic review of tobacco smoking behaviour among young persons aged 10 to 24 years revealed prevalence rates of 0.2% to 32.5 %^14^. In addition, previous studies have shown that between 26.0 % and 66.0 % of in-school adolescents in Nigeria drink alcohol^15^,^16^. Globally, unhealthy diets low in vegetables/fruits and high in sugar and fatty contents increase the risk of cardiovascular diseases^17^. Evidence from previous studies in Nigeria have revealed high consumption of sugary beverages, pastries as well as low fruits/vegetables among in-school adolescents^18^,^19^.

Nigeria currently has an estimated population of over 200 million people^20^, and adolescents constitute over 20% of the population^21^,^22^. Therefore, interventions to address NCDs risk behaviours among adolescents will ultimately reduce the economic burden of NCDs in Nigeria in the future. Sociodemographic factors, such as age, gender, income, and education, play a pivotal role in NCD risks. Lower income and education levels have been shown to correlate with higher NCD risk, highlighting the importance of addressing sociodemographic and socioeconomic determinants of health.^2^,^3^ There are limited data on the co-occurrence and pattern of multiple NCDs risk behaviours especially among the adolescent population in Nigeria. It is also not clear whether the sociodemographic predictors of multiple NCD risk behaviours among adolescents in Nigeria is comparable to those in middle- and high-income countries. Thus, there is a need for more studies on multiple NCDs risk behaviours among this important sub-group in the Nigerian population. Thus, this study was conducted to assess the prevalence and sociodemographic predictors of multiple NCD risk behaviours among Nigerian in-school adolescents. The findings will provide the scientific basis for targeted interventions tailored towards in-school adolescents in the study setting and other similar settings in Nigeria.

## Methods

### Study setting, design, and participants

The study setting was at nine secondary schools randomly selected across the three senatorial districts of Delta State in the oil-rich Niger Delta region of Nigeria. Delta State is a culturally diverse state with a total of 758 secondary schools with a total population of 262,242 students distributed across the three senatorial districts^23^. The study design was a school-based cross-sectional survey carried out between December 2021 and May 2022 among a random multistage sample of apparently healthy students aged between 10 and 19 years enrolled in secondary schools in Delta State, Nigeria. The Health Research Ethics Committee (HREC), Delta State University Teaching Hospital provided ethical clearance for the study (HREC/PAN/2021/016/0326). The study followed relevant guidelines and regulations. The authorities of the selected schools provided access permission before the commencement of the study. Participation in the survey was without coercion or inducement. The right to withdraw participation in the survey without untoward consequences was made known to the study participants and their parents/guardians. The study participants who were aged less than 18 years provided assent, while those aged 18 years and above provided verbal informed consent to participate in the study. Written informed consent was obtained from their parents/guardians.

### Sample size determination and sampling procedure

Fisher's formula was employed to determine the minimum sample size. Based on an estimated prevalence of 50% for multiple NCD-related risk behaviours among secondary school students in Nigeria, an error margin of 5 % and a standard normal variate at a 95% confidence level, the minimum sample size was estimated at 384 students. However, 607 students participated in the study.

Sample selection was in two stages. In the first stage, a table of random numbers was used to select nine secondary schools (three per senatorial district) from a sampling frame of 758 secondary schools in Delta State. In the second stage, the total number of students in each class level and their sex distribution was determined across the nine selected secondary schools. The percentage of students in each sex-class combination was calculated and proportionately allocated into sex strata by their class levels in each school to allow for adequate and equitable representation of both sexes within each class level. A table of random numbers was then used to randomly select students based on their number in the register in each class level by sex strata across the nine selected secondary schools.

### Study instrument and data collection

Data were collected using an interviewer-administered semi-structured questionnaire adapted from the WHO STEPS questionnaire^24^. On the scheduled days of visit to the different selected schools, trained data collectors used the questionnaire to elicit information on the sociodemographic characteristics, and NCDs risk behaviours of the study participants. NCDs risk behaviors were assessed by questions on cigarette smoking, alcohol consumption, diet/nutritional habits, and physical activity habits.

### Outcome and independent variables

The outcome variables were NCDs risk behaviours among the study participants. NCDs risk behaviours were assessed by questions on cigarette smoking, alcohol consumption, unhealthy diet consumption, and physical inactivity habits. Study participants who self-report ed that they currently smoke cigarettes were considered as smokers, while those who previously smoked or never smoked cigarettes were considered as non-smokers. Study participants who self-reported that they currently consume alcohol were considered as consumers of alcohol. In contrast, those who previously consumed alcohol or who have never consumed alcohol were considered as non-consumers of alcohol. Study participants who self-reported a daily intake of fewer than five servings of fruits/vegetables (raw or cooked) or regular high intake of saturated fat or sugary meals/drinks were categorised as consumers of unhealthy diets. Study participants who self-reported less than 150 minutes/week of moderate to vigorous-intensive physical activity (walking, recreational exercise, cycling) were considered to have inadequate/insufficient physical activity^25^. The study participants were classified as having no risk, one risk, two co-current risks, three co-current risks, and four co-current risks according to the number of risk behaviors they self-reported.

The independent variables include age, sex, place of residence, family socioeconomic status and mothers' educational attainment. Family socioeconomic status was determined using the scoring scheme for classifying socioeconomic status in Nigeria. The scoring scheme takes into cognisance both parents' educational attainment and occupation^26^.

### Statistical analyses

Data collected was analysed using the IBM SPSS version 22 software. Descriptive and inferential analysis was carried out. Categorical variables were summarised as frequencies and percentages (summarised data were presented in tables and figures). Bivariate and multivariate analysis using Pearson's chi-square and multinomial logistic regression respectively was carried out. The 95 % Confidence Intervals (CI) and p-values obtained was reported in two tail form and statistical significance determined at p-value less than 0.05. The multinomial logistic regression was performed to identify predictors of the outcome variable of interest (multiple NCDs risk behaviours). All variables significant during bivariate analysis using Pearson's chi-square at a p-value < 0.2 were entered stepwise into the multinomial regression model to obtain the Adjusted Odds Ratio (AOR) of each factor on the outcome variable at 95 % confidence interval.

## Results

The total number of study participants was 607, all of whom were included in the analyses.

### Sociodemographic characteristics of the study population

The mean age of the study participants was 14.7 (SD = 1.52) years, 54.0 % (n = 328) of which were aged 15 – 19 years, and 46.0 % (n = 279) were aged 10 - 14 years. Over half of the study participants (57.2 %: n = 347) were females, compared to their male counterparts (42.8 %: n = 260). Over half of the study participants (56.2 %: n = 341) were urban residents, compared to their rural counterparts (43.8 %: n = 266).

The highest proportion of the study participants' families belonged to the middle socioeconomic stratum (68.7%: n=417), compared to those whose families belonged to the lower (18.6%: n=113) and upper (12.7%: n=77) socioeconomic stratum (SES) respectively. The highest proportion of the study participants' mothers had secondary education (55.8%: n = 339) compared to those whose mothers had primary (24.1%: n=146) and tertiary (20.1%: n=122), education respectively (Table 1).

**Table 1:** Sociodemographic characteristics of the study participants

Variables	Categories	N = 607 (%)
Age (Years)	10 - 14	279 (46.0)
	15 - 19	328 (54.0)
**Mean age ± SD (years)**	14.7 ± 1.52 years	
Sex	Male	260 (42.8)
	Female	347 (57.2)
Place of residence	Urban	341 (56.2)
	Rural	266 (43.8)
Mothers' education	Primary	146 (24.1)
	Secondary	339 (55.8)
	Tertiary	122 (20.1)
Family socioeconomic status	Upper	77 (12.7)
	Middle	417 (68.7)
	Lower	113 (18.6)

### Prevalence of NCDs self-reported risk behaviour among the study population

**Figure 1:**
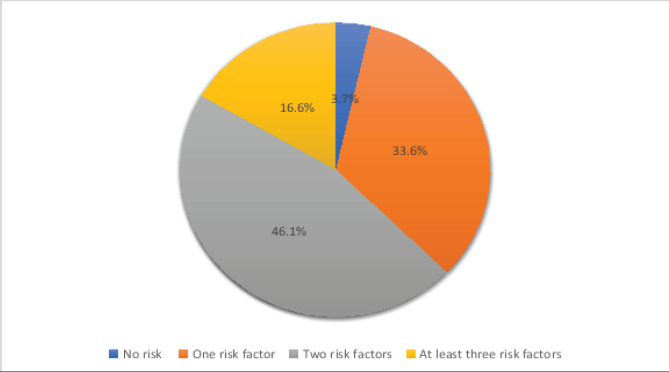
Proportion of the study participants with no risk, one risk, and multiple NCD risk behaviours

**Figure 2:**
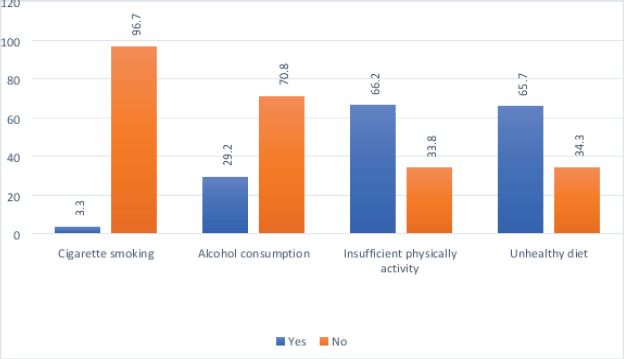
Prevalence of NCD risk behaviours among the study participants

**Figure 3:**
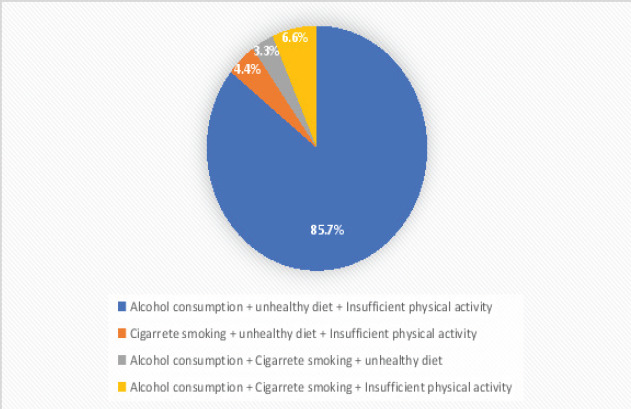
Clusters of three co-occurring NCDs risk behaviours among the study participants

**Figure 4:**
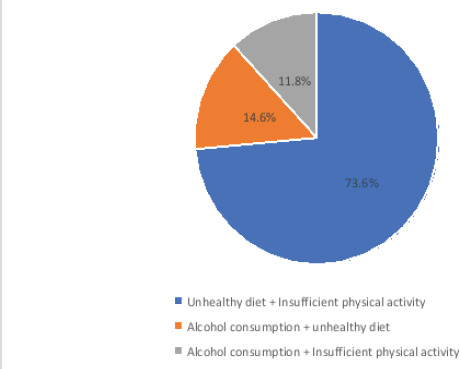
Clusters of two co-occurring NCDs risk behaviours among the study participants

The prevalence of two and at least three co-occurring NCDs risk behaviours among the study participants is 46.1 % (n=280) and 16.6 % (n = 101) (Figure 1). Among the study participants, 3.3% (n = 20) self-reported that they were current cigarette smokers, 29.2% (n = 177) self-reported current alcohol use, 66.2% (n=402) self-reported inadequate physical activity, and 65.7% (n=436) self-reported consumption of unhealthy diet (Figure 2). Among the study participants with three co-occurring NCDs risk behaviours, 85.7 % (n = 78) of them had clustering of alcohol consumption, unhealthy diet consumption, and insufficient physical activity (Figure 3). Among the study participants with two co-occurring NCDs risk behaviours, 73.6 % (n =206) of them had clustering of unhealthy diet consumption and insufficient physical activity (Figure 4).

### Sociodemographic predictors of multiple risk behaviours among the study population

There was a statistically significant association of study participants' age (*χ*^2^ = 9.27; df = 3; P = 0.026), sex (*χ*^2^ = 14.54; df = 3; P = 0.002), and place of residence (*χ*^2^ = 24.61; df = 3; P < 0.001) with the occurrence of multiple NCDs risk behaviours (Table 2).

**Table 2:** Association of socio-demographic factors with occurrence of multiple NCDs risk factors among the study participants

Variables	Total number 607 (100%)	No risk 22 (3.7%)	One risk 204 (33.6%)	Two risks 280 (46.1%)	Three to four risks 101 (16.6%)	Bivariate Analysis (Chi-Square)
**Age Group (Years)**						
10 - 14	279 (46.0)	14 (5.0)	92 (33.0)	138 (49.5)	35 (12.5)	*χ*^2^ = 9.27; df = 3; P = 0.026
15 - 19	328 (54.0)	8 (2.4)	112 (34.2)	142 (43.3)	66 (20.1)	

**Sex**						
Male	260 (42.8)	7 (2.7)	78 (30.0)	115 (44.2)	60 (23.1)	*χ*^2^ = 14.54; df = 3; P = 0.002
Female	347 (57.2)	15 (4.3)	126 (36.3)	165 (47.6)	41 (11.8)	

**Place of residence**						
Urban	341 (56.2)	8 (2.3)	90 (26.4)	180 (52.8)	63 (18.5)	*χ*^2^ = 24.61; df = 3; P < 0.001
Rural	266 (43.8)	14 (5.3)	114 (42.8)	100 (37.6)	38 (14.3)	

**Mothers' education**						
Primary	146 (24.1)	7 (4.8)	54 (36.9)	62 (42.5)	23 (15.8)	*χ*^2^ = 4.84; df = 6; P = 0.565
Secondary	339 (55.8)	9 (2.7)	109 (32.2)	159 (46.9)	62 (18.2)	
Tertiary	122 (20.1)	6 (4.9)	41 (33.6)	59 (48.4)	16 (13.1)	

**Family socioeconomic status**						
Upper	77 (12.7)	2 (2.6)	27 (35.1)	35 (45.5)	13 (16.8)	*χ^2^ = 2.74; df = 6; P = 0.841
Middle	417 (68.7)	13 (3.1)	140 (33.6)	194 (46.5)	70 (16.8)	
Lower	113 (18.6)	7 (6.2)	37 (32.7)	51 (45.2)	18 (15.9)	

* Fisher's test

Clustering of two simultaneous NCDs risk behaviours was mainly observed among study participants who were urban dwellers. Compared to their rural counterparts, study participants who were urban dwellers had two-fold increase odds of adopting two simultaneous NCDs risk behaviours (AOR=1.86; 95% CI: 1.34 - 2.57) (Table 3). Clustering of at least three simultaneous NCDs risk behaviours was mainly observed among study participants who were aged 15-19 years. Compared to their counterparts (10-14 years), study participants who were aged 15-19 years had two-fold increase odds of adopting at least three simultaneous NCDs risk behaviours (AOR=1.84; 95% CI: 1.11 – 3.05) (Table 3). In addition, study participants who were male had two-fold increase odds of adopting at least three simultaneous NCDs risk behaviours (AOR=1.75; 95% CI: 1.28 – 2.82) (Table 3). Furthermore, study participants who were urban dwellers had 41 % increase odds of adopting at least three simultaneous NCDs risk behaviours (AOR=1.41; 95% CI: 1.06 – 2.86) (Table 3).

**Table 3:** Predictors of multiple NCD risk factors among the study participants

Variables	Two risk factors		At least three risk factors	

	Multinomial Logistic Regression		Multinomial Logistic Regression	
	AOR (95% CI)	P-value	AOR (95% CI)	P-value
**Age (Years)**				
10 - 14			1	
15 - 19	-	-	1.84 (1.11 - 3.05)	0.018

**Sex**				
Male	-	-	1.75 (1.28 - 2.82)	0.023
Female			1	

**Place of residence**				
Urban	1.86 (1.34 - 2.57)	0.009	1.41 (1.06 - 2.86)	0.039
Rural	1		1	

## Discussion

The prevalence of multiple NCDs risk behaviours was relatively high among the study participants. This is a source for concern as risk behaviours adopted during adolescence tend to remain in adulthood and have been shown to contribute to two-thirds of NCDs premature mortality in adult populations^27^. The clustering of two co-occurring NCDs risk behaviours was more prevalent compared to the clustering of three or more co-occurring NCDs risk behaviours. This observation however contrast the findings from previous studies conducted among adolescents in Brazil^28^ and Curacao^29^ which revealed that clustering of three or more co-occurring NCDs risk behaviours were more prevalent.

Among the study participants with three or more co-occurring NCDs risk behaviours, the cluster of alcohol consumption, unhealthy diet consumption, and insufficient physical activity was prevalent. The clustering of unhealthy diet consumption and insufficient physical activity was prevalent among the study participants with two co-occurring NCDs risk behaviours. Insufficient physical activity and unhealthy diet consumption were prevalent NCDs risk behaviours among the study participants. This is a source of concern as both insufficient physical activity and unhealthy diet consumption have been shown to increase the risk of NCDs. Conversely, both healthy dietary patterns and physical activity have been shown to be consistently associated with reduced risk of the occurrence of hypertension, heart diseases, stroke, obesity, hyperlipidaemia, and diabetes^30^-^32^.

The undue emphasis on intellectual pursuit at the expense of physical activity, fitness, and sports in school settings in Nigeria leaves a lot to be desired. Presence of well managed sports fields and facilities in public and private schools seems to be a thing of the past. In addition, the insidious nature of the social media and availability of internet enabled hand-held devices also reduce the quality and quantity of time available for school adolescents to exercise and engage effectively together in sports. Furthermore, unavailability of adequate sports facility and infrastructure at community level also limits opportunities for physical activity among adolescents in their spare time.

Alcohol use among the study participants was quite substantial and this may not be unconnected with the consequence of peer influence and/or increased availability of alcoholic beverages. A critical issue is the current sale of alcoholic drinks, especially gins and whiskey, in small volumes (sachets) making it increasingly affordable and readily available to adolescents. Despite the primordial preventive measures by the government that have significantly reduced advertisement of alcoholic beverages in the mass media in Nigeria, adolescents are still taking to consumption of alcohol. Inappropriate alcohol use could also be the gateway to illicit drug use, hence preventive interventions should target adolescents to ensure they do not uptake this habit in the first place.

Sociodemographic characteristics such as study participants' place of residence, age, and sex were associated with the occurrence of multiple risk behaviours. Study participants who were urban residents had two-fold increased likelihood and forty-one percent increased odds of having two and at least three co-occurring NCDs risk behaviours respectively. This observation is consistent with findings from previous studies which have shown that adolescents who are urban dwellers are more likely to adopt NCDs risk behaviours compared to their rural counterparts^7^,^8^. Consistent with the findings from previous studies,^29^,^33^-^36^ this study found that older age increased the likelihood of the co-occurrence of multiple NCDs risk behaviours. Older adolescents (15-19 years) had a two-fold increase likelihood of having multiple risk behaviours compared to younger adolescents (10-14 years). This may not be unconnected with the fact that older adolescentsare less dependent on parental support and are more likely to be influenced by peer pressure toward adopting risk behaviours than younger adolescents. Also consistent with the findings from previous studies,^29^,^33^-^36^ this study found that male adolescents had a two-fold increase likelihood of having multiple risk behaviours compared to female adolescents. These sex differences may not be unconnected with social conditions surrounding the concepts of identity that are inherent in males and females. Evidence has shown that the concepts of identity and environmental factors is associated with gender-specific preference and choice with regards to NCDs risk behaviours among adolescents^36^.

In this study, no association was observed between mothers' level of education, and family socioeconomic status with the occurrence of multiple NCDs risk behaviours among the study participants. This observation contrasts the findings from previous studies which have revealed that mothers' level of education as well as family socioeconomic status play a role in shaping the co-occurrence of unhealthy behaviors among adolescents^37^-^39^. Lower maternal education have been shown to limit access to health information and resources and may contribute to adolescents adopting multiple risk behaviors^38^. Socioeconomic factors play a role in shaping risk behaviors by mediating the relationship between maternal education and risk behaviors among adolescents^39^.

The findings of our study should be interpreted considering the following strengths and limitations. The main strength of our study is that it targeted a relatively larger population of in-school adolescents. However, our study has some limitations: firstly, the self-report nature of NCDs risk behaviours by the students leaves room for reporter bias. Secondly, this study employed a cross-sectional descriptive design; thus, it is not possible to determine either causality or directionality of the variables analysed.

## Conclusion

The result of this study indicates a relatively high prevalence of multiple NCDs risk behaviours among the study participants; and increasing age, male sex, and being urban residents were the sociodemographic predictors of the co-occurrence NCDs risk behaviours. The findings from this study calls for urgent implementation of interventions at all ecological levels that will improve adolescents' NCDs risk knowledge and equip them with the skills to adopt healthy lifestyles and choices. This will consequently deter their engagement in unhealthy behaviours. We recommend further strengthening of school health education and school health service components of the National School Health Programme to improve information and communication on NCDs risk.
